# Exploring System Context Contributing to Emerging REDD+ Collaborative Governance Regime in Ghana: Stakeholders Perceptions at the National Level

**DOI:** 10.1007/s00267-024-02085-y

**Published:** 2024-11-21

**Authors:** Misharch Kwadwo Osei

**Affiliations:** Department of Political Science, Umeå University, Umeå, Sweden

**Keywords:** REDD+, Collaborative governance, System context, Drivers of collaboration, Ghana, Qualitative analysis

## Abstract

Since its emergence in 2007, the global mechanism for Reducing Emissions from Deforestation and Degradation in developing countries (REDD+) has raised hopes of providing cost-effective solutions to climate change. However, the design and implementation of REDD+ projects in many developing countries, including Ghana, have faced complex governance challenges. In recent years, a collaborative governance approach has been increasingly recommended for effective REDD+ implementation, but the impact of the dynamics of developing countries’ context on collaboration success remains unclear. Using Ghana’s Cocoa Forest REDD+ Programme (GCFRP) as a case study, this paper aims to increase our understanding of how the dynamics of developing countries’ context affect the drivers shaping the initiation of REDD+ collaborative regimes for transforming cocoa forest landscapes. Through qualitative content analysis of document reviews and semi-structured interviews with national program stakeholder groups, the results indicate that Ghana’s dynamic context facilitates collaboration on REDD+ implementation when stakeholders feel uncertain about the future availability of forest resources and recognize their interdependence in responding to such issues. Additionally, the findings of the study indicate that strong political will for change, along with strategic windows of opportunity created by REDD+ funding mechanisms, play a vital role in shaping consequential incentives essential for aligning stakeholder interests and fostering cross-sector leadership for initiating the REDD+ collaborative governance regime. While the applicability and limitations of the IFCG framework are discussed, further in-depth studies at project levels are crucial to understanding local stakeholders’ perspectives on the key elements necessary for successful collaboration.

## Highlights

- Establishment of REDD+ collaborative governance in Ghana is qualitatively explored.
- Diverse system context factors influence REDD+ collaborative governance regime.
- Multiple stakeholders have collaborated in it in Ghana’s High Forest Zone.
- Implications for REDD+ policies and projects in developing countries are discussed.
- The IFCG framework is used and its applicability is highlighted.

## Introduction

The REDD+ mechanism, established under the auspices of the United Nations Framework Convention on Climate Change (UNFCCC), serves as a significant governance instrument for climate change mitigation. It seeks to incentivize forest-rich developing countries to reduce emissions from deforestation and forest degradation while also providing additional benefits such as sustainable forest conservation and management and socio-economic development for local communities, thereby contributing to the broader goals of sustainable development (Simonet et al. 2020). However, weak system contexts of forest and land use pose persistent challenges to the establishment and implementation of REDD+ initiatives in many participating developing countries (Dwisatrio et al. 2021; Kengoum et al. 2020; Korhonen-Kurki et al. 2014; Loft et al., 2017). From the outset, REDD+ governance faces significant challenges in ensuring meaningful stakeholder engagement, as diverse interests and power dynamics often marginalize vulnerable communities, undermining initiative legitimacy (Cadman et al. 2017; Duchelle et al. 2018; Pham et al. 2021). Furthermore, fragmented governance structures and complex decision-making procedures have impeded efficient multi-level coordination, resulting in inefficiencies and conflicting priorities among various authorities (Rodriguez-Ward et al. 2018; Wunder et al. 2020). In addition, REDD+ has been criticized for concerns over exacerbating land-tenure conflicts and inequities in benefit distribution among stakeholders (Awung and Marchant 2020; Duchelle et al. 2018; Dawson et al. 2018; Milne et al. 2019). Moreover, persistent uncertainty and unreliability in funding sources have hindered project development, scalability, and long-term sustainability (Morita and Matsumoto 2023). In light of the significant challenges facing REDD+ governance, there is a growing call for innovative governance strategies to effectively address these issues to ensure the long-term sustainability of REDD+ activities (Angelsen et al. 2018; Turnhout et al. 2017).

In recent years, collaborative governance, where a broad spectrum of stakeholders with conflicting interests in REDD+ projects including government agencies, private sector actors, environmental NGOs, CSOs, and local communities work in partnership to jointly design, implement, and manage REDD+ initiatives, is increasingly seen as an effective strategy to tackle the multifaceted challenges in REDD+ governance (Angelsen et al. 2018; Furumo and Lambin 2020; Roengtam and Agustiyara 2022; Shin et al. 2022). A response to dealing with REDD+ complexity and various policy design and governance challenges exemplifies the broader shift toward collaborative natural resource management (Bodin 2017; Jager et al. 2020). This approach has gained prominence in recent decades, driven by the recognition that environmental issues like deforestation, biodiversity loss, climate change, and water scarcity are inherently complex, interconnected problems often spanning multiple jurisdictions and affecting diverse stakeholders, that traditional top-down governance approaches, where government agencies solely control natural resource management, have frequently failed to achieve sustainable outcomes (Bodin 2017; Bodin et al. 2020; Jager et al. 2020; Newig et al. 2018; Ulibarri et al. 2020). The growing focus on the application of collaborative approaches in natural resource governance has led to the emergence of various interdisciplinary, transdisciplinary, and systems-oriented frameworks, including Ostrom’s Institutional Analysis and Development (IAD) framework and the general framework for analyzing the sustainability of social-ecological systems (Ostrom 2005, 2009), adaptive co-management (Folke 2006), the polycentric governance approach (e.g., Nagendra and Ostrom 2012; Carlisle and Gruby 2019), and the interactive governance approach (Kooiman et al. 2008), among others (for an overview, see Partelow et al. 2020), all of which collectively aim to contribute to a more nuanced understanding of governing complex natural resource systems.

Building on and synthesizing many of these systems-oriented approaches and empirical research, Emerson et al. (2012) and Emerson and Nabatchi (2015) developed one of the most comprehensive and adaptable frameworks for studying collaborative governance: the Integrative Framework for Collaborative Governance (IFCG). The IFCG is a versatile system approach that has been applied across diverse fields, including environmental management, public health, urban planning, and economic development in diverse contexts (Emerson and Nabatchi 2015; Ulibarri et al. 2023). The IFCG conceptualizes collaborative governance as a governance system in which *“the processes and structures of public policy decision-making engage people across the boundaries of public agencies, levels of government, and or the public-private and civic spheres to carry out a public purpose that could not otherwise be accomplished*” (Emerson et al. 2012, p. 2; see also Emerson and Nabatchi 2015 p. 18). The IFCG outlines that the system^1^ context influences the essential drivers that activate the initiation of a collaborative governance regime (CGR). In turn, collaboration dynamics produce actions that result in tangible outcomes and adaptations throughout the system (Emerson et al. 2012; Emerson and Nabatchi 2015).

The IFCG specifically underscores that a CGR develops within a system context in response to the complex and evolving nature of challenges that encourage stakeholders to devise collective solutions (Emerson et al. 2012). This system context refers to a broad range of dynamic surrounding conditions, including “*public resources or service conditions, legal and policy frameworks, socioeconomic and cultural characteristics, network characteristics, political dynamics and power relationships, and history of conflicts*” that create opportunities and constraints for initiating and sustaining the CGR (Emerson and Nabatchi 2015, p. 41). As a result, Emerson and Nabatchi (2015) emphasize that understanding the role of the system context is crucial, given that collaborative governance is fundamentally embedded within it. The prevailing conditions can either foster opportunities for or constraints on the emergence of CGR, thereby influencing their processes and performance over time. The IFCG has been widely applied to explore the system context and outcomes of different complex natural resources management in varied cultural, political, and geographical settings (e.g., Dressel et al. 2020; Johansson 2024; Sandström et al. 2013; Thellbro et al. 2018). Nevertheless, these studies exhibit a pronounced geographical bias, with a significant concentration on case studies from advanced developed democratic countries, notably in North America, Northern, and Western Europe (Douglas et al. 2020; Ulibarri et al. 2023). This raises concerns about the generalizability of the IFCG (Ulibarri et al. 2023), particularly regarding its applicability to the governance processes within the complex and multifaceted contexts of natural resource management in developing countries. Moreover, while existing research suggests that many CGRs emerge in response to complex environmental challenges that necessitate cross-sector collaborative governance involving a diverse range of state and non-state actors (e.g., Imperial 2023; Imperial et al. 2016; Ulibarri et al. 2020), they can clearly take different forms and be organized in a variety of ways. In line with Ulibarri et al.’s (2023, p. 501) assertion, more research is warranted to investigate how contextual differences shape the drivers behind CGR formation to better manage the turbulent period surrounding the establishment of new collaborations.

To assist efforts to meet this need, an exploratory analysis of how the particular dynamics of developing countries’ context may affect the success of collaboration on REDD+ implementation in Ghana, a REDD+ participating country, is presented here. Ghana provides an excellent setting for such a case study due to its abundant forest resources and troublingly high deforestation rate, which is currently estimated at 3.2% per annum and one of the highest in the world (FAO 2020; Nukpezah and Alemagi 2020). Ghana’s economic development and the livelihoods of its smallholder communities are heavily reliant on agriculture, particularly cocoa production which accounts for approximately 4% of the national Gross Domestic Product (GDP) and provides livelihoods for approximately six million people, or roughly 30% of the country’s population (Arhin 2015; Olwig et al. 2024). However, recent economic growth, driven primarily by the cocoa industry, has had detrimental impacts on the country’s forest resources and land degradation, particularly across the cocoa forest landscapes in the High Forest Zone (HFZ) (Brobbey et al. 2020; Government of Ghana 2016; Nasser et al. 2020; Olwig et al. 2024). The HFZ, which is part of the West African Guinean Forest biodiversity hotspot, is distinguished by its dense tropical rainforests and abundant biodiversity, which are critical for conservation strategies and carbon sequestration efforts associated with the global REDD+ initiative (Carodenuto 2019; FAO 2020; Nukpezah and Alemagi 2020). Ghana is also one of numerous sub-Saharan African countries facing persistent challenges associated with high poverty rates, especially in rural areas where the sustainability of livelihoods strongly depends on access to, and utilization of, natural resources (World Bank 2022). Furthermore, despite recent decentralization efforts, the country’s governance structure is still centralized, with weak administrative capacities at regional and district levels to effectively implement REDD + policies and projects (Akamani et al. 2015; Ameyaw et al. 2016). Moreover, the implementation of local REDD + pilot initiatives has often marginalized local community interests, leading to conflicts and power struggles between government bodies, corporations, and local communities (Appiah et al. 2015; Saeed et al. 2018; Soliev et al. 2021). These challenging system context conditions may impose considerable limitations on the effectiveness of collaborative natural resource management and conservation initiatives (Akamani et al. 2015). Amid the tumultuous context of Ghana’s weak forest governance system, the establishment and implementation of REDD+ CGR must contend with persistent challenges in adapting to dynamic processes to achieve sustainable social and ecological outcomes (Akamani et al. 2015).

In this article, the IFCG is used to analyze the effects of the particular dynamics of the system context of forest governance in Ghana’s HFZ on the specific drivers for establishing REDD+ CGR. The specific research questions addressed are: (1) How applicable is the IFCG framework in the context of REDD+ programs in developing countries? (2) What conditions are essential to sustain a collaborative REDD+ governance approach that effectively benefits both the environment and local communities in developing countries? The paper is structured as follows. The next section presents the IFCG, including potentially influential system context factors that may promote or hinder the initiation and sustenance of CGRs, thereby influencing their processes and performance. The following sections describe the research design and data collection processes, present the results, and finally discuss the findings in the light of theoretical expectations.

## Theoretical Framework

As stated in the introduction, the analytical foundations for this study are provided by the IFCG, which conceptualizes a collaborative governance regime (CGR) as being embedded or nested in a broader system context. Various system context factors may drive (or hinder) the CGR’s initiation. Within the CGR, collaborative dynamics give rise to outputs and actions that iteratively generate outcomes and potential adaptations (Emerson and Nabatchi 2015). The framework’s comprehensiveness facilitates in-depth investigation of the establishment, implementation, and resulting actions and outcomes of collaboration. The IFCG framework is deemed highly suitable for this study due to its inherent systems approach, flexibility, comprehensiveness, and clear distinction between contextual factors and specific drivers for the initiation of collaborative governance (Emerson et al. 2012; Biddle 2017; Thellbro et al. 2018; Ulibarri et al. 2023). This allows for the analysis of the impact of system context on specific drivers for the initiation of the REDD+ collaborative governance regime for achieving a reduction of deforestation and sustainable cocoa production in the HFZ.

According to the IFCG, the specific drivers for CGR initiation include *leadership* (the presence of an identified leader who facilitates the initiation process by providing resources and other necessary support); *consequential incentives* (identified societal or organizational importance); *interdependence* (perceptions that problems are beyond a single organization’s ability to address); and *uncertainty* (reduction or sharing risks in addressing complex problems) (Emerson and Nabatchi 2015). A CGR is initiated and shaped by the presence of at least one or more of the four specific drivers (Emerson and Nabatchi 2015). Moreover, the IFCG specifies two system-specific characteristics: the nature of the focal policy issue and the structure of the deciding authorities, which affect whether the CGR will be “*self-initiated*,” “*independently convened*,” or “*externally directed*” (Emerson and Nabatchi 2015, p. 19). Due to space limitations, this study examines the influence of resource conditions, policy frameworks, socioeconomic and cultural characteristics, political dynamics and power relations, and history of conflict on the drivers of the initiation of REDD+ CGR projects in Ghana, as briefly discussed below.

*Resource conditions* here refer to the biophysical characteristics of public forest resource provision in Ghana’s high forest zone. These are explored through analysis of interviewed participants’ perceptions of the state of public forest resources, such as the high rate of deforestation and degradation, depletion of environmental quality and potential pressures or risks of forest-resource shortages, current and future risks of climate change and the extent of risks to provision of benefits related to forests’ use such as carbon sequestration, cultural, or socioeconomic reliance on them.

*Policy and legal frameworks* refer to the existing forest policies and how or why they are established (Emerson and Nabatchi 2015). This is captured through interviewed respondents’ perceptions of the extent to which collaborative decision-making and efforts are enabled or constrained by current legal and policy frameworks, with consideration of policy changes that have already been initiated in the forestry land use sector.

*Socioeconomic and cultural characteristics* embrace a complex set of potentially relevant factors related to developmental status, including educational levels, income, health, race, and ethnic diversity (Emerson and Nabatchi 2015, p. 188). Here, they are investigated through interviewed respondents’ perceptions of natural resource dependency in the area, poverty levels, population density, and the extent to which these socioeconomic factors are linked to deforestation and influence stakeholders’ participation in the CGR.

*Political dynamics and power relations* encompass the degrees of access to power and inclusion of local actors in decision-making, political stability or polarization, democratic values, and access to and distribution of power by various stakeholders affected by a policy problem (Emerson and Nabatchi 2015). Here, these contextual elements are explored through interviewed respondents’ expressed perceptions of forestland tenure arrangements, actors, jurisdictions, and institutions involved in forest and land management, and the extent to which power relations emanating from their interactions affect multiple actors’ commitment and motivation to participate in collaborative forest governance endeavors (Emerson and Nabatchi 2015).

The *history of conflicts* refers to perceived levels of (mis)trust, disagreements, or struggles before the emergence of the collaboration process (Emerson and Nabatchi 2015). Here, this is explored through interviewed respondents’ perceptions of the dominant forest conflicts, the factors driving these conflicts, who is involved, the consequences of forest conflicts, previous failures to address them, and the extent to which these factors affect collaboration in REDD+ programs.

## Methodology and Materials

**Fig. 1:**
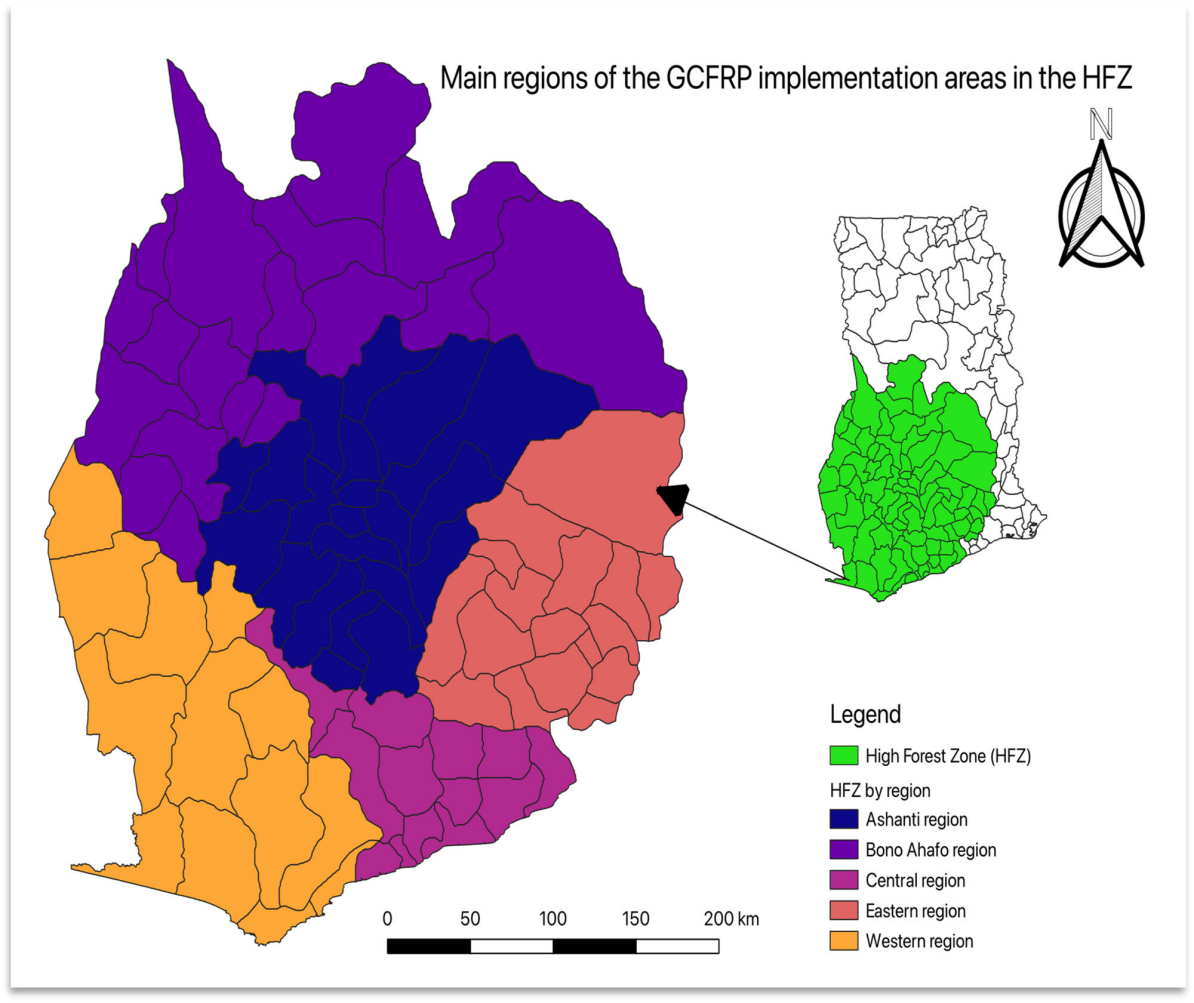
Map of Ghana showing the five administrative regions within the HFZ designated as priority areas for GCFRP implementation. (Source: Adapted from Government of Ghana 2017)

Qualitative single-case methodology was applied together with descriptive analysis (Yin 2018) to obtain empirical data for this study. The case study approach was selected because it is particularly suitable when the researcher has limited influence over the focal subject or the primary objective is to address contemporary, real-world phenomena (Yin 2018). The analysis focused on the Ghana Cocoa Forest REDD+ Programme (GCFRP), which was launched in 2017 as the first global commodity-based REDD+ initiative (Government of Ghana 2017; Johnson 2021). The GCFRP represents Ghana’s flagship emission reduction initiative, designed to realize the goals of its REDD+ strategy and promote sustainable cocoa production in the country (Government of Ghana 2016). Within the HFZ, this initiative covers an area of approximately 5.92 million hectares encompassing almost 92 administrative districts spread over five regions, namely Ashanti, Eastern, Central, Western, and Brong-Ahafo (see Fig. 1). The GCFRP is a multi-level CGR initiative that involves the collaboration of important government agencies like the Forestry Commission and Ghana Cocoa Board, working with civil society organizations (CSOs), private cocoa companies, environmental nongovernmental organizations (ENGOs), and local communities to design and implement national REDD+ program activities. The initiative aims to reduce deforestation, boost cocoa farm resilience, produce climate-smart cocoa, and improve farmers’ livelihoods across the cocoa forest landscape (Government of Ghana 2017). The GCFRP is emerging within Ghana’s geographically dynamic and contested natural resources management contexts of the HFZ and thus makes it an instrumental case to validate, extend, and or adjust the IFCG framework.

This study employed a combination of qualitative methods, including the analysis of project documentation and conducting semi-structured interviews. It commenced with a systematic content analysis of key policy and project documents such as Ghana’s REDD+ Readiness Preparation Proposal, Ghana’s REDD+ Strategy, and the GCFRP development and implementation plan report. These documents covered institutional arrangements, engagement principles, social and environmental safeguards, and forest reference levels and monitoring. This initial phase aimed to establish the project background, stated objectives and goals, and the problem it aims to address strategies, and implementation plans (see Appendix 1 for a comprehensive list of reviewed documents). Following the project documentation analysis, ten semi-structured interviews with key informants from various national stakeholder groups were carried out using the snowball sampling method. These participants included representatives from specified government bodies (e.g., Forestry Commission and Ghana Cocoa Board), environmental non-governmental organizations, civil society, and the private sector (see Table 1 for an overview of interviews). The decision to exclude local stakeholders affected by GCFRP and instead focus on national-level stakeholders was made to capture the initiation process of the GCFRP where these stakeholders were central. The interviews began with an initial contact via email to the Climate Change Directorate of the Forestry Commission, which houses the National REDD+ Secretariat (NRS) and oversees the implementation and monitoring of program activities under GCFRP. Building on this initial correspondence and utilizing my professional connections within Ghana’s REDD+ governance, I identified and reached out to key informants for the study. All interviews were conducted in English at the participants’ workplaces, using a pre-developed interview protocol (see Appendix 2) that included questions based on the different themes of the IFCG framework to elicit detailed responses from the participants. Each interview lasted between 45 and 60 min. All interviews were tape-recorded with respondents’ written consent and transcribed verbatim for content analysis using Microsoft Word’s automated transcription function.

**Table 1:** Overview of interviewees from national stakeholder groups

Stakeholders	Type of organization	Number of interviews	Code
Forestry Commission (FC)	Government Agency	2	GA-01; GA-02
Ghana Cocoa Board (COCOBOD)	Government Agency	2	GA-03; GA-04
Nature Conservation Research Centre (NCRC)	Civil Society Organization	1	CSO-01
Solidaridad West Africa Network	Civil Society Organization	1	CSO-02
Proforest Ghana	Environmental Non-governmental Organization	1	EN-01
Tropenbos Ghana	Environmental Non-governmental Organization	1	EN-02
A Rocha Ghana	Environmental Non-governmental Organization	1	EN-03
World Cocoa Foundation	Private Sector	1	PS-01
Total		10	

The data were analyzed using theory-driven qualitative content analysis (Fisher and Aguinis 2017; Kuckartz 2019), for which QSR International NVivo software version 2020 (QSR International 2020) was used. The data analysis employed both inductive and deductive approaches and findings were presented using quotations. Initially, an inductive approach was applied to evaluate the raw data to discover patterns and generate preliminary themes and a set of codes based on the system context and driver components of the IFCG (see Appendix 3). Then a deductive approach was applied to sort the data into the specified set of codes to interpret and contextualize these findings. Although the study focuses on stakeholders’ perceptions of the effects of system context factors on collaboration success, it did not involve the collection of sensitive personal data. Thus, it did not meet the need for ethical review by the Swedish Ethical Review Authority (https://etikprovningsmyndigheten.se). Nevertheless, I strove to apply good research practices in accordance with the Swedish Research Council’s recommendations (Swedish Research Council 2017) throughout the study to ensure the confidentiality and ethical integrity of the work, including the establishment of voluntary consent. These practices included anonymization of the respondents, as shown in Table 1.

## Results

This section consecutively presents results about each of the five sets of system context variables, then an analysis of their impacts on drivers of the REDD+ CGR’s initiation.

### System Context Elements

#### Resource conditions

According to nine of ten respondents, the state of forest resources in the HFZ has deteriorated significantly as a result of the alarming rate of deforestation, posing significant climate change threats to cocoa production sustainability and the ability of REDD+ projects to meet their emission reduction targets. The tenth respondent perceives the current state of forest resources as both a challenge and an opportunity for the implementation of REDD+ projects highlighting that the forest resources in the HFZ serve multiple purposes for various stakeholders and are currently facing significant deforestation and degradation, largely driven by agricultural expansion and illegal logging. This alarming rate of deforestation trend has led to a notable decline in biodiversity and ecosystem services, raising serious concerns among stakeholders about the long-term sustainability of these forest resources. The HFZ encompasses approximately 5.79 million hectares (around 12.4 million acres) of dense tropical rainforests, making it a vital biodiversity hotspot for conservation efforts. Additionally, this area holds significant potential for REDD+ projects aimed at achieving emission reduction targets (Government of Ghana 2010, 2017; COCOBOD 2020). Situated in the southwestern region of Ghana, the HFZ spans five key administrative regions: Ashanti, Brong-Ahafo, Eastern, Western, and Central regions. The HFZ which serves as Ghana’s most productive agroecological zone for high-value cocoa cash-crop production, is also critical to the country’s biodiversity, carbon storage, and overall environmental health, supporting a variety of ecosystems and local community livelihoods (Government of Ghana 2016; NCRC 2020; Olwig et al. 2024; Proforest Ghana 2021). However, their perspectives on the causes of the changing resource conditions vary, acknowledging that there are discrepancies among respondents regarding the causes of deforestation and land degradation. For example, government officials attributed the primary drivers of deforestation in the HFZ to unsustainable land use practices within cocoa farming, such as extensive clearing of forests for new plantations. GA-01 explains:

“The single most important driver of deforestation in the HFZ is unsustainable farming practices driven by the expansion of cocoa production. Deforestation poses a significant danger to biodiversity and forest ecosystem services, and if we do not act to safeguard remaining forests and restore degraded ecosystems and their services, there will be fewer carbon sinks and therefore more emissions.”

Conversely, the private sector within the cocoa industry highlights the connection between deforestation and the ever-changing dynamics of the global cocoa market which creates insecurity and uncertainty for farmers about their future income. In response, farmers may clear more land for cocoa cultivation to increase production and buffer against potential income losses during periods of low prices. This expansion into forested areas is the leading cause of deforestation as highlighted by PS-01:

“You know Ghana’s cocoa is sold exclusively on the international market, leaving us vulnerable to fluctuating prices and market uncertainties that drive farmers to clear forests for expanded cocoa cultivation for more income, intensifying deforestation. To ensure cocoa production remains profitable and environmentally responsible, we must unite to implement comprehensive policies that address these challenges.”

**Fig. 2:**
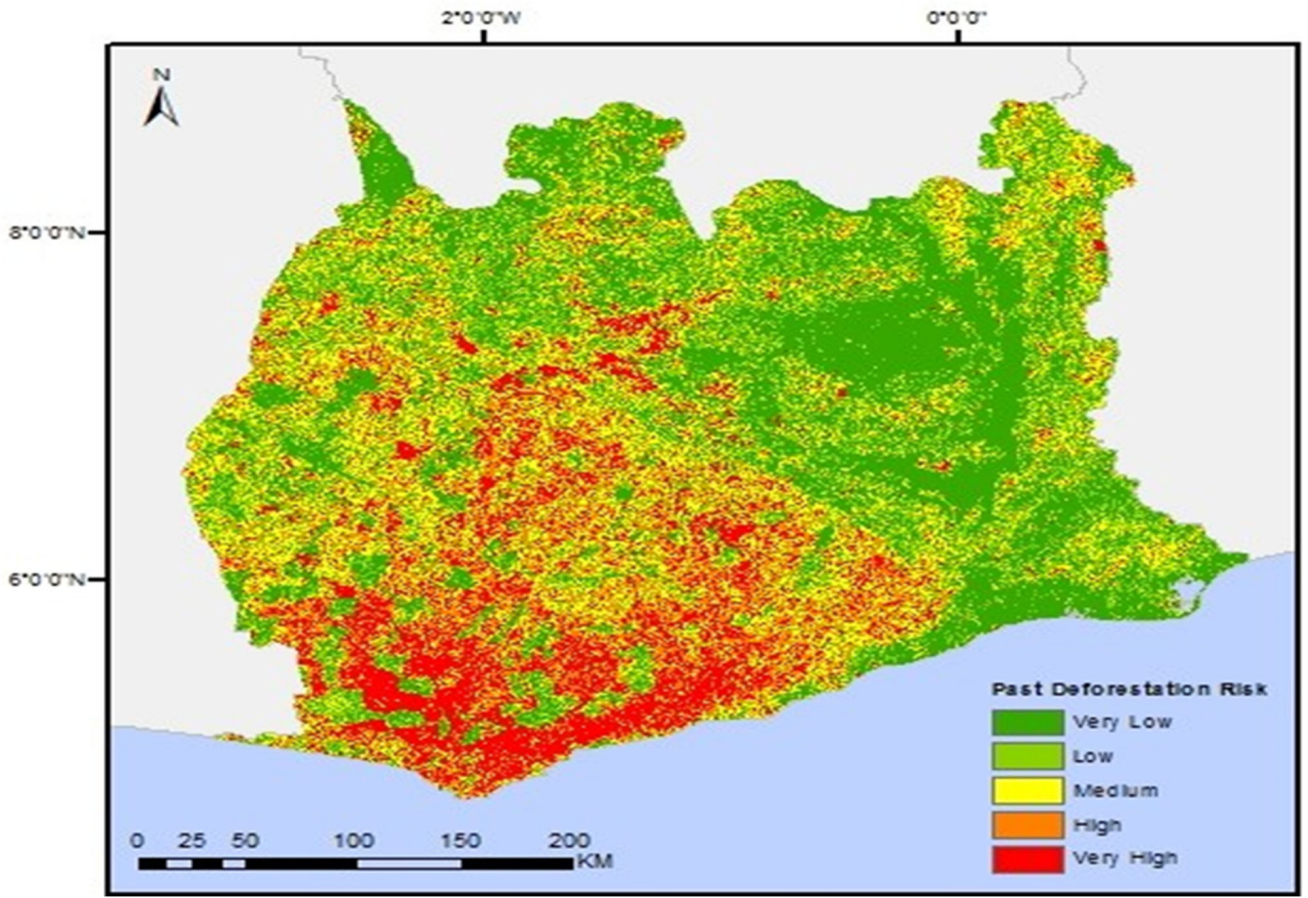
Forest cover map of Ghana showing rates of deforestation in the GCFRP implementation areas in HFZ in the years 2000–2015, ranging from very low (green) to very high (red). Adapted from the Government of Ghana (2017b). [Accessed on 5 June 2023]

These quotations illustrate how almost all respondents (*n* = 9) perceived that the HFZ forest is deteriorating, with negative effects on the current and future provision of forest ecosystem service functions, including the region’s enormous forest carbon sequestration capacities and multiple actors’ socioeconomic benefits from forest resources. They also highlight views that REDD+ projects can help to tackle, cost-effectively, high deforestation rates in the HFZ (illustrated in Fig. 2), reduce emissions, and promote sustainable development. Conversely, the respondents recognized that deforestation could exacerbate resource scarcity, by causing (*inter alia*) reductions in carbon stocks, timber supplies, clean water, and non-timber forest products, with negative impacts on diverse stakeholders, including local communities, governments, environmental organizations, and industries. The findings strongly indicate that deforestation poses a substantial threat to numerous stakeholders. As these critical forest ecosystem services decline, the urgency for collaborative efforts in REDD+ implementation becomes increasingly evident. Stakeholders recognize that preserving and restoring forest ecosystems is essential not only for mitigating climate change but also for sustaining cocoa production and the livelihoods that depend on these vital resources. This awareness fosters a collaborative approach to REDD+, where diverse actors unite to develop and implement strategies that protect and enhance forest ecosystem services, ultimately contributing to a more sustainable and resilient future.

### Policy and Legal Frameworks

The interview findings shed light on how the ongoing devasting rate of deforestation and climate change as shown in the resource conditions is related to policy and legal framework factors related to natural resource management in Ghana, revealing how policy inconsistencies, weak enforcement, and limited stakeholder involvement before the GCFRP exacerbate the problem. The perspectives shared by private sector representatives as “… *the challenge we face in Ghana is that our cocoa and forestry policies often pull in opposite directions. While cocoa farming is encouraged for economic growth, forestry policies aim to curb deforestation […]. This creates a conflict, as expanding cocoa farms frequently leads to clearing forests. There is a lack of integrated planning, and until both sectors can align their goals, deforestation will continue to undermine our sustainability efforts*.” *(PS-01)*.

Respondents representing ENGOs highlight the disconnect between policy design and local realities. This misalignment often results in weak policy enforcement and a failure to address the needs of communities that rely on forest resources for their livelihoods. As one ENGO representative expressed, “*Top-down policies often look good on paper but fail in practice when they ignore the voices of those most affected local communities. When enforcement is weak and local realities are overlooked, policies meant to protect forests become ineffective. Without genuine engagement and recognition of local people’s needs and knowledge, deforestation remains an ongoing consequence of these disconnected approaches*.” *(EN-01)*. This view was echoed by another informant who stated: *“One of the most pressing challenges is the absence of genuine engagement with local communities, forcing smallholders to clear forests for cocoa farming and driving deforestation to critical levels. Unless we embrace inclusive decision-making to tackle these deep-rooted issues, the fight against deforestation in Ghana’s cocoa sector will continue to falter.” (EN-03)*. Similarly, insights from CSO representatives point to governance challenges such as ambiguous land tenure rights and policies favoring large corporations over forest preservation. CSO-02 conveyed this sentiment: *“While it might be convenient to blame smallholders as the usual suspects, Ghana’s biggest problem lies in uncertain regulations, corruption, and policies that favor large corporations over forest preservation.”*

The document analysis complements these findings by tracing the origins of the development of Ghana’s REDD+ Strategy, which initiated policy reforms aimed at promoting cross-sector, participatory, and collaborative approaches to address the complexities and challenges of sustainable forestry and land-use management in the HFZ. The findings reveal that Ghana’s REDD+ readiness preparation phase marked a crucial period of policy reform, with a strong emphasis on promoting inclusive participation as a central pillar for achieving sustainable forest management and deforestation reduction (Government of Ghana 2016). During this phase, the government recognized the complexities of natural resource governance in the HFZ and realized that the goals of REDD+ could not be achieved without first addressing underlying fundamental fragmented policies and legal frameworks governing natural resource management (Asare and Kwakye 2013; Government of Ghana 2016). An analysis of the literature on Ghana’s REDD+ policy process and the implementation of early pilot projects (e.g., Asare 2013; Asare and Kwakye 2013; Asiyanbi et al. 2017; Den Besten et al. 2019; Johnson 2021; Nukpezah and Alemagi 2020) reveals that both international climate change initiatives and national forestry legislation emphasizing participation and engagement from a diverse array of stakeholders, including local communities have spurred collaboration REDD+ programs in HFZ. The analysis indicates that the drafting of the national REDD+ strategy prompted substantial reforms aimed at addressing the complex challenges related to forest management and carbon emissions. Among these reforms, tree and land tenure reforms, legislation on carbon rights, and benefit-sharing mechanisms emerged as foundational elements. They were perceived not merely as technical modifications but as strategic priorities crucial for establishing conditions that promote multi-stakeholder participation and inclusivity, with a specific emphasis on engaging local communities in REDD+ initiatives (Asare 2013; Asare and Kwakye 2013; Government of Ghana 2016). This indicates that the GCFRP is deeply embedded within the framework of Ghana’s National REDD+ Strategy (2016–2035), which emphasizes the necessity of a collaborative cross-sectoral approach. Additionally, it demonstrates strong alignment with other pertinent national policies addressing forestry, land use, and climate change mitigation and adaptation. At the national level, the analysis reveals that the GCFRP is firmly grounded in the 1992 Constitution of the Republic of Ghana, which mandates the government to protect forests and conserve biodiversity while implementing measures for their restoration in the event of degradation. Furthermore, Ghana’s Shared Growth and Development Agenda I and II delineates the responsibilities of various governmental agencies and sectors in the management of environmental and natural resources. These, along with the National Climate Change Policy (2013), National Forest and Wildlife Policy (2012), National Gender Policy (2015), Cocoa Sector Development Strategy I (1999), Timber Resources Management Act 617 (Amendment) Act, 2002, and the Forest and Plantation Development Act, 2000 (Act 583), collectively offer an enabling environment for multi-stakeholder engagement and foster cross-sectoral and multi-level coordination in the development and implementation of Ghana’s national REDD+ initiatives (Government of Ghana 2016, 2017). Despite the positive impact of existing policy and legal frameworks on cross-sector collaboration and participation in REDD+ activities, some respondents highlighted the Forest and Wildlife Policy (2012) as a pivotal milestone in Ghana’s REDD+ forest management and biodiversity conservation. This policy demonstrated early signs of sectoral interconnectedness in addressing issues in both agriculture and forestry, including the marginalization of local communities, land tenure insecurity, and inequalities in benefit distribution which drive deforestation and unsustainable utilization of forest and land resources. Additionally, market-driven mechanisms were introduced under this policy to incentive community participation and enhance the sustainable use of forest resources, as exemplified by EN-01:

“Ghana’s REDD+ Strategy and the GCFRP are rooted in favorable policy contexts and anchored relevant national policies and strategies, such as the National Forest and Wildlife Policy, which establish a strong foundation for multistakeholder collaboration on REDD+.”

This demonstrates that Ghana’s REDD+ Strategy, which underpins the establishment and implementation of the collaborative project, aligns with several important international climate change treaties, including the UNFCCC Paris Agreement. It also aligns with national forestry legislation, particularly the National Climate Change Policy (2013) and National Forest and Wildlife Policy (2012), which have provisions that promote cross-sector collaboration among relevant actors and sectors for REDD+, such as forestry and agriculture. The GCFRP collaborative effort is thus congruent with the global REDD+ institutional architecture to combat deforestation and climate change through a multisectoral collaborative governance strategy.

#### Socioeconomic and cultural characteristics

In terms of socioeconomic and cultural characteristics, the document analysis revealed that the HFZ area is home to over twenty million people, 75% of Ghana’s population (Government of Ghana 2018, 2019a; Proforest Ghana 2021). Respondents indicated that many of these people live in rural communities with low levels of education and heavily depend on forest resources and subsistence agriculture. As already described, nine of ten respondents think that deforestation and land degradation are challenging issues in the HFZ, affecting various natural resource users and management groups both economically and politically. The interviews revealed that the livelihoods of most people residing in the region have heavily depended on agricultural intensification. However, widespread rural poverty causes people to rely on subsistence farming, with encroachment and clearance of forestland for expansion of cocoa cultivation to feed their families. Eight of the ten respondents clearly express that socio-economic issues are highly relevant regarding the forest land use management system in the HFZ and strong enabling factors of the collaborative project’s initiation. The motivations of ENGOs and government agency respondents are illustrated by the following two quotes:

“We are interested in conserving our forests not just by preventing illegal logging, mining, and illicit agricultural expansion, but also in selling carbon credits and using the proceeds to improve forest management and alleviate rural poverty.” (*EN-03)*.

“Deforestation is often a symptom of deeper socioeconomic issues such as poverty and lack of infrastructure. When communities are struggling to survive, they may resort to unsustainable practices. By improving local economies and providing viable alternatives, we can help reduce the reliance on deforestation for survival” (PS-01).

The analysis of the interviews revealed that multiple stakeholders are highly dependent on forest resources for their livelihood. Therefore, forests play crucial roles in livelihood diversification in the HFZ. Socioeconomic factors such as high population density, low education levels, and high poverty rates have significantly influenced deforestation and land degradation. Moreover, the government has limited resources to counter these problems. Thus, the findings of this study collectively underscore the importance of addressing socioeconomic factors to enable the success of REDD+ projects.

#### Political dynamics and power relations

Data from the interviews and document analysis revealed that political dynamics and power relations were integral contextual conditions that have played important roles in shaping the initiation of REDD+ CGR in the HFZ. Natural resource management in Ghana has had a checkered history, with cycles of centralization and decentralization (Asare 2013; Asare and Kwakye 2013; Teye 2011; Interview EN-02). Under the British colonial authority, all forest resources in Ghana held in common by traditional authorities became the property of the state. This tendency continued in independent Ghana, with the Forestry Commission in charge of managing all forest reserves on behalf of the government. However, outside of designated reserves, forests are owned and managed by individual and community owners, while all off-reserve tree resources are legally vested in the state in trust for the owners. Almost all the respondents (90% (*n* = 9)) said that this complicated structure of overlapping and fragmented claims of rights between state forest departments and customary forms of forest ownership and tenure systems in Ghana has caused complex power relations between multiple actors who have vested interests in forest resources. Their perceptions indicate that interactions between social actors and their interests have influenced management outcomes through processes such as decision-making, compliance with rules and conventions, law enforcement, and forest practices. For example, the CSO respondents indicated that the conservation of natural resources is actively supported by all levels of government. Consequently, Ghana’s approach to sustainable natural resource management has historically been driven by the central government, utilizing protected forest reserves and implementing conservation programs through a top-down policy-making approach. They argued that this top-down exclusionary approach has posed significant problems and controversies. As a consequence, the protected forest reserves in the HFZ have experienced extensive deforestation due to the expansion of cocoa and crop farming and other human activities such as illegal mining, chain-saw operations, and charcoal production. Many communities, both within and outside protected forest reserves, depend on these areas for their livelihoods and cultural practices. However, while the government labels these groups as illegal encroachers based on their legal rights to access, use, and manage natural resources in these regions, local communities often perceive state control as obstructive and unfavorable which threaten their rights and access to forests. Talking about this issue, a respondent from the CSO group said:

“The exclusion of local communities from forest management not only endangers the ecosystem but also damages these communities’ social fabric and cultural heritage” (CSO-02).

The ENGO respondents rather perceive that government policies favoring large-scale agricultural investments often disregard the rights of local communities and small farmers. This creates a power dynamic where corporate interests overshadow the voices of those directly impacted by land grabbing and environmental degradation, as captured by EN-01:

“We’ve seen how policies incentivizing large-scale agriculture lead to extensive deforestation and environmental degradation. Smallholder farmers continue to face considerable challenges competing with large-scale agricultural enterprises mostly from abroad that benefit from preferential access to subsidies and under government-backed initiatives. These practices not only harm biodiversity but also displace forest-dependent communities, exacerbate inequalities, and limit opportunities for smaller producers.”

The respondent representing the private sector shared a similar view:

“Government policies and industry practices must prioritize empowering local communities with access to decision-making processes. This inclusion is critical for addressing issues like deforestation, ensuring sustainable cocoa production, and respecting community rights.” (PS-01).

On the contrary, respondents of government agencies highlight concerns about the unintended consequences of relying heavily on international donor funding for environmental and natural resource conservation that comes with conditional agreements or donor funding requirements that may prioritize certain management approaches. They emphasize that while international donor funding plays a vital role in supporting conservation initiatives in Ghana, it has reinforced the centralization of decision-making and prioritization of technical expertise. This dynamic often excludes local communities with weaker capacities from meaningful participation. This concern was captured by GA-04 as:

“You know in Ghana international donor funding has been instrumental in advancing environmental conservation efforts [but] in most cases funding projects prioritize external technical solutions which inadvertently sidelined local communities with weak capacities from meaningful participation. It’s crucial we strike a balance that integrates local knowledge, empowers and builds local community resilience for effective conservation outcomes.”

The findings of this study, illustrated by these quotations, clearly indicate that the government and powerful groups have previously controlled the decision-making process and/or outcomes of forest management. Stakeholders from the ENGO sector stressed that power imbalances are reflected in government policies and legal frameworks that support large-scale agricultural investments over forest conservation and smallholder livelihoods. Six of the seven non-governmental respondents expressed that the governance and management arrangements for natural resources often favor industry interests, which leads to the exploitation of resources without consideration of the long-term impacts on local communities and ecosystems. The respondents collectively identified historical power dynamics and inequities rooted in colonial forest governance as significant underlying causes of deforestation. One government official noted, “… *it’s clear that top-down management has failed us […] We need a collaborative approach that includes local voices and recognizes their knowledge and experiences[…] Only then can we address the deep-rooted issues driving deforestation” (GA-02)*. Such insights highlight a consensus among stakeholders that addressing these challenges requires a fundamental shift away from traditional top-down management strategies towards a more inclusive governance model. The study thus revealed that political dynamics and power inequalities were critical factors in fostering a conducive environment for the initiation of REDD+ CGR in the HFZ.

#### History of conflicts

The analysis revealed that the HFZ has hosted several REDD+ pilot and environmental conservation projects and their implementation has raised numerous issues associated with justice and histories of conflict. These contextual factors have engendered numerous disputes regarding land use between government agencies, private companies or REDD+ promoters, and local populations. The findings indicate that the management of natural resources in the HFZ is frequently a hotspot for conflict, with contestation over land boundaries, and both access to and exploitation of forest resources being common in this area. Nine of the ten respondents unanimously agreed that the interplay of tree and land tenure insecurity, inequitable benefit-sharing structures, powerful political and economic interests, and cultural values has created a complex landscape in which conflicts arise, often exacerbating Ghana’s REDD+ forest governance challenges. In addressing the complex challenges of deforestation, respondents emphasized that land tenure reform, equitable benefit-sharing, and the establishment of transparent institutions are crucial. As one respondent stated, “… *without clear land tenure rights, cocoa farming communities will always feel insecure about their investments in forest conservation […] We need tenure reforms and carbon rights legislations that will ensure local people have ownership and control over the forestland they depend on.” (CSO-01)*. Another participant highlighted the importance of equitable benefit-sharing, noting*, “… it’s essential that the benefits from REDD+ initiatives are shared fairly. If local communities do not see tangible benefits, their engagement will dwindle, and so will the effectiveness of conservation efforts.” (PS-01)*. Additionally, insights revealed significant barriers, including the exclusion of community members from meaningful project participation. Two government officials remarked:

“The increasing demand for agricultural land, along with issues of land tenure insecurity, has historically been a source of tension and competing claims over land in the HFZ. Addressing these challenges requires a collaborative approach to create comprehensive solutions that meet the concerns of local communities, environmental groups, and businesses including the underlying land tenure security issues.” (GA-01).

“… we often underestimate the value of local knowledge […]. Excluding community members from the decision-making process not only breeds resentment but also undermines the success of the projects.” (GA-03).

Respondents also expressed concerns about the inability to meet the high expectations of local communities. As one respondent representing the private sector pointed out*, “Communities engaging in [REDD+] and other environmental conservation projects expect results quickly, but many of these projects take time to show benefits[…]. We need better communication and realistic timelines to manage these expectations effectively.”(PS-01)*. Competing interests and insufficient coordination among government agencies regulating both the forestry and cocoa sectors were also identified as critical challenges. Two respondents representing ENGOs stated:

“Tackling forest conflicts requires a form of collective effort if we can win the war against deforestation. It requires more than just political party manifestos and sweet slogans; they require proactive participation of both private and local community stakeholders.” (*EN-02)*

“There is often a lack of alignment between different agencies […]. If we want to tackle deforestation effectively, we need a unified approach that considers both the forestry and cocoa sectors together.”(EN-03).

These quotations collectively demonstrate a unanimous recognition among respondents of the detrimental impact that conflicts have on rising deforestation rates and the sustainable implementation of REDD+ projects, highlighting the critical role that addressing these conflicts plays as a driver that facilitated the emergence of REDD+ CGR. As a result, the historical context of land use conflicts within natural resource management in the HFZ underscored the necessity to try collaborative governance to handle conflicts and promote inclusive decision-making. Together these results provide important insights into how conflicts become a driver that is crucial for initiating the GCFRP. These findings illustrate that the historical context of conflicts in forest management not only shapes the challenges faced in addressing deforestation but also fosters the emergence of REDD+ CGR by highlighting the need for inclusive decision-making, equitable benefit-sharing, and transparent institutions that empower local communities and recognize their invaluable contributions to sustainable forest management (Table 2).

**Table 2:** A summary of the keywords of system context elements influencing the initiation of REDD+ CGR in Ghana’s HFZ

System context elements	Keywords related to system context conditions necessitating the initiation of REDD+ CGR
Resource conditions	• High rates of deforestation and degradation • Resource overexploitation • Agriculture expansion (cocoa farming expansion into forested areas) • Unsustainable cocoa farming practices • Loss of biodiversity • Climate change impacts • Depletion of carbon stock • Soil and water quality decline affecting cocoa yields
Policy and legal frameworks	• Supportive international Climate change Agreement (Paris Agreement) • National Climate Change Policy and REDD+ regulations • Community-based forestry policies • Climate-smart agriculture policies • Cocoa sector regulations such as deforestation-free cocoa commitments.
Socioeconomic and cultural characteristics	• Population growth • Dependence on single-crop agriculture • High rates of poverty • Economic vulnerability • Low-income levels • Policy failures to address rural poverty • High dependence on forest resources • Lack of access to education
Political dynamics and power relations	• Conflicting regulatory mandates between government agencies • Fragmented and unsecured land tenure and carbon rights for cocoa farmers • Inequitable distribution of benefits • Marginalization of rural communities in decision-making processes • Corruption and elite capture in project management • Lobbying by powerful industry stakeholders
History of conflicts	• Land tenure conflicts • Benefit-sharing conflicts • Cocoa production versus conservation conflicts • Historical grievances and unmet rural people expectations from REDD+ pilot/environmental conservation projects • Long-term sustainability concerns • Community rights violations

### Contextual Conditions Impact on Drivers of Collaboration

The analysis indicates that the national context of the forest and land use system was ripe for the evolution of collaborative REDD+ governance in the HFZ. This encompasses elements from all the dimensions described among the system context conditions of the IFCG (see section “Theoretical framework”). The depletion of forest resources and extensive deforestation has given rise to shared environmental concerns, and economic and social pressures, and created a collective sense of uncertainty and interdependency among various stakeholders in the agriculture and forestry sectors. The context is grounded in enabling policy and legal frameworks that enable multistakeholder collaboration on REDD+. However, political dynamics and significant power imbalances within local communities and across various levels of governance present significant challenges and are evident in the earlier inadequate efforts towards sustainable forest management. Moreover, widespread poverty and weak socioeconomic development contribute to a high degree of historical conflict among stakeholders, which has created competition for limited resources and fostered mistrust. In this context, all four essential drivers (*uncertainty, interdependence, consequential incentives, and initiating leadership*) have emerged for the establishment of REDD+ CGR The respondents particularly noted uncertainty in the shared economic and climate change risks was clearly present. The findings revealed that HFZ areas are important for cocoa production and endowed with forest resources that could potentially retain more carbon and hence support REDD+ implementation, but there are also high rates of deforestation in them. Hence, there is substantial uncertainty about the future provision of resources and the potential effects on many actors. CSO-01 explained this as follows:

“Deforestation is a major driver of climate change but the intense competition for forest lands in the HFZ for diverse purposes puts its carbon reduction targets under threat.”

This reveals a perceived risk of a scarcity of crucial forest resources for the program to meet their emission reduction targets and cocoa production.

Interdependence, the second identified driver of collaboration, is closely intertwined with uncertainty. The findings revealed that the forests in the HFZ play vital roles in invaluable ecosystem services and sustaining cocoa production (the bedrock of the Ghanaian economy and rural livelihoods). However, unsustainable cocoa farming expansion by smallholders has threatened the functionality of forests in the HFZ. The uncertainty has necessitated cross-sector sectoral collaboration and interactions between the agriculture and forestry sectors when implementing the GCFRP activities, as explained by PS-01:

“The long history of conflict between various government departments and local communities has resulted in more extensive and severe deforestation […]. Cocoa and deforestation are very complex issues that one organization cannot handle […]. We need to move from doing things in silos and form partnerships and do things together to make changes.” (PS-01).

Seven of the respondents made similar reflections, recognizing that the complexities of forest management cannot be overstated as it requires resources and knowledge that no single actors, or specific groups of sectors, can access on their own are needed to address challenges facing the forestry and cocoa sectors. Hence, collaboration with other parties, including local communities, is needed to resolve associated governance challenges. A respondent representing CSOs reiterated this perspective, stating*, “… the sustainability of local livelihoods is intricately linked to the health of forest ecosystems […]. If the forest is destroyed, the soil loses its richness, leading to a decline in cocoa production.” (CSO-02)*. This view was echoed by a government official who noted that “… *we must involve local communities in decision-making processes, as they depend on forest resources for their livelihoods […]. Ignoring their voices not only risks conflicts but also undermines the very conservation efforts we aim to achieve” (GA-03)*. Similarly, an ENGO representative stated, “… the health of our forests is directly linked to the well-being of local communities*[…]*. *If we want to avoid conflicts over resources and effectively tackle the challenges of deforestation and climate change, it’s crucial to involve these communities in the decision-making process and recognize their rights.”(EN-03)*. In accordance with Zachrisson and Beland Lindahl (2013), the interviews illustrate that a weaker party usually initiates conflicts to enforce interdependence on a stronger party. These varied perspectives underscore that people are motivated to collaborate only when key actors recognize interdependencies according to Emerson and Nabatchi (2015, p. 45–46). Furthermore, without adequate processes for resolving conflict at the local level, policies to tackle deforestation and climate change may reproduce power differentials, further marginalize some groups, and fuel further conflict. The findings of this study confirm that while positive past experiences with cooperation are likely to foster support for future collaborative endeavors, longstanding conflicts can also act as motivators, particularly when stakeholders are out of options and recognize that collaboration is the only viable approach for addressing issues (Zachrisson et al. 2018).

Furthermore, the interdependence of forestry and cocoa interests in REDD+ activities, as well as the uncertainty created by the high rate of deforestation and pressure on forest resources, has provided consequential incentives for actors to collaborate. Most respondents recognized the significance of the consequential incentives in this case, which have allowed different parties to collaborate in REDD+ implementation. They particularly recognized that poor socioeconomic conditions, power inequalities, and a long history of conflict are the primary causes of high deforestation rates, which negatively affect resource conditions and generate uncertainty. As a result, the collaborative project aligns with stakeholders’ shared interests in leveraging REDD+ finance and other resources to address forestry and cocoa governance issues in the HFZ, thereby countering deforestation and promoting rural socio-economic development. EN-02 illustrated this as follows:

“The GCFRP program aligns with these goals, providing incentives for reducing deforestation and emissions, tackling challenges in rural development, and combating climate change.”

The respondents’ comments suggest that the collaborative project may have been partly driven by the parties’ perceptions of the benefits of addressing context-related problems. Perceived benefits include improvements in forest resource conditions, incentives for smallholder farmers to implement climate-smart cocoa production practices, and the sustainability of supply chains of agricultural commodities. Finally, the results show that the FC and COCOBOD provided essential leadership through effective facilitation and coordination, which was key to the establishment of the GCFRP, particularly by promoting the commitment of all interested parties to participate in the collaborative initiative. CSO-01 explained:

“The super fantastic commitment and environment created by the Forestry Commission and COCOBOD are the reasons it all happened. We had leaders in government, the specific people who were behind us. These were the types of personalities who were open to input, who were good leaders, and who were not holding territory. We needed sort of champions and we had a champion in the Forestry Commission and COCOBOD. They created an environment in which all these different stakeholders could come and so we had very good energy and relationships.”

## Concluding Discussion

The IFCG was employed in this study to analyze the effect of the dynamic system context of weak forest governance in developing countries on the essential drivers for initiating a REDD+ CGR in Ghana. The turbulent context of weak governance has long been a significant barrier to effective collaborative conservation and sustainable natural resource management in developing countries, including Ghana (Agrawal and Gupta 2005; Akamani et al. 2015). This analysis indicates that these challenges have also fostered an environment conducive to the emergence of collaborative governance strategies, particularly within the REDD+ mechanism. Insights from national stakeholders suggest that collaborative governance provides a pathway to address the complexities of forest and cocoa governance by promoting inclusive decision-making processes that involve a diverse array of stakeholders, including governmental bodies, civil society organizations (CSOs), private sector companies, environmental non-governmental organizations (ENGOs), and local communities. In contrast to the contexts of advanced democratic countries, where significant emphasis is placed on preexisting trust, social capital, and established networks (e.g., Emerson and Nabatchi 2015; Dressel et al. 2020; Johansson 2024; Mancheva 2018; Mattor and Cheng 2015; Sandström et al. 2013; Thellbro et al. 2018), findings from this research indicate that in developing countries the reversed conditions such as limited institutional capacities, pervasive land-use conflicts, poverty, and corruption together with the complex challenges posed by high deforestation rates and climate change, can drive collaborative governance. These contextual conditions generated uncertainties, interdependencies, and consequential incentives, and then the state also provided cross-sectoral leadership, which collectively have acted as essential drivers triggering the initiation of REDD+ CGR.

The characteristics of the GCFRP as promising to become a multi-level CGR, along with evidence of how the identified drivers shaped its formation, align with the IFCG framework’s classification of “externally directed” CGR (Emerson and Nabatchi 2015). Such regimes are typically initiated by state actors or agencies with well-defined authority to tackle broad, recurring policy challenges within specific sectors, employing either mandatory participation or incentive-based approaches (Emerson and Nabatchi 2015; Ulibarri et al. 2023). The GCFRP is widely recognized as a “pioneering example” and an “undeniable success story” of a REDD+ initiative, characterized by a multi-stakeholder, cross-sectoral approach to transforming cocoa forest landscapes, which has successfully secured $50 million in funding for a 10-year implementation plan aimed at improving forestland use governance, with strong support from national and international stakeholders (FCPF 2021; Government of Ghana 2017; NCRC 2020; Proforest Ghana 2021). Despite these superlatives of the government of Ghana and other non-government stakeholders, the GCFRP, as an externally-driven CGR, faces the daunting challenge of counteracting the pressures from narrow political and sectoral interests at higher levels to achieve sustainability outcomes (Bruun and Rubin 2023; Emerson and Nabatchi 2015; Ulibarri et al. 2023). In the multi-level policy settings of REDD+, the implementation success of the GCFRP hinges on formalizing and developing these promising national policies and institutional structures to actually delineate guidelines for local collaborative initiatives. This will include well-defined stakeholder roles and accountability mechanisms, reforms in land and tree tenure rights, and the implementation of equitable benefit-sharing mechanisms, all of which are critical for fostering local stakeholder engagement and sustainability outcomes (Angelsen et al. 2018; Boyd et al. 2018; Brown 2018; Ros-Tonen et al. 2018; Rodriguez-Ward et al. 2018; Wunder et al. 2020; van der Haar et al. 2023). Furthermore, empowering local communities to determine and influence their conservation needs has been integral to the success of the application and sustainability outcomes of collaborative governance in climate change mitigation and natural resource management initiatives particularly significant in developing countries like Ghana, where local livelihoods are heavily reliant on natural resources and agriculture (Bastakoti and Davidsen 2017; Koomson 2024a, b; Pham et al. 2021). Moreover, the success of the GCFRP relies heavily on secure, long-term funding, which is crucial for the effective implementation of REDD+ initiatives. Without stable financial support to establish robust community-based governance structures to incentivize sustainable agricultural practices among local communities, as well as to monitor and evaluate progress, these efforts may falter, ultimately undermining the overall effectiveness of the programs (Morita and Matsumoto 2023; Shin et al. 2022).

The Ghanaian case study illustrates the utility of the IFCG framework as a useful analytical lens for deepening our understanding of the system context as a critical condition in fostering collaborative REDD+ governance in developing countries. This research not only corroborates but also expands upon the findings of Emerson (2018) and Hayter and Nisar (2018), highlighting that, amid the challenges of weak governance in developing countries, collaborative governance emerged as an instrumental approach employed by government and policymakers to align stakeholder interests and collectively address complex environmental and socioeconomic challenges. In this light, this study responds to the call from Ulibarri et al. (2023) for further investigation into how the unique dynamics of developing countries shape the emergence and success of collaborative natural resource management. Yet, while the analysis of the GCFRP highlights that the challenging context of natural resource management systems in the HFZ created an excellent environment that fosters the emergence of collaborative REDD+ governance, it also exposes a notable limitation of the IFCG framework in relation to REDD+ implementation in developing countries. Specifically, the analysis indicates that the IFCG framework does not adequately account for two critical factors: the critical role of securing high-level national political will and the importance of a window of opportunity in spurring stakeholder collaboration on REDD+ implementation.

First, addressing large-scale, commodity-driven deforestation and sustainability challenges in Ghana, as in other countries, necessitates comprehensive governmental intervention at the national level. This includes developing national action plans and implementing policy reforms to tackle entrenched sectoral interests in land use while strengthening forest laws and enforcement capabilities to effectively mitigate emissions drivers originating from multiple sectors and levels beyond the forestry sector (Wurtzebach et al. 2019; van der Haar et al. 2023). The lessons from the GCFRP case highlight that strong commitment and political will from the Government of Ghana, particularly from key state institutions such as the FC and COCOBOD has been crucial for the successful initiation and implementation of the REDD+ initiative through a cross-sector collaborative governance approach. Second, the GCFRP’s success is partly due to the favorable window of opportunity created in the context of Article 6 of the Paris Agreement, which permits both public and private actors to engage in REDD+ programs to earn carbon credits to meet their climate commitment goals.

In conclusion, this research has provided important theoretical and practical insights by exploring the unique dynamics within the context of Ghana and their influence on the emergence of a REDD+ CGR. It contributes to the wider literature on collaborative natural resource management and underscores the significance of contextual variables in determining the success of collaboration (e.g., Bodin 2017; Cockburn et al. 2020; Dressel et al. 2020; Emerson and Nabatchi 2015; Swette et al. 2023; Zachrisson and Beland Lindahl 2013). While the GCFRP represents a significant step forward in the development of REDD+ CGRs, uncertainty remains about whether these approaches will ultimately improve outcomes for both communities and the environment, especially in Ghana’s developing contexts. Future research should focus on in-depth critical exploration of local stakeholders’ perspectives to document the processes and challenges involved in establishing and implementing local collaborative REDD+ projects to comprehensively contribute to our understanding of the performance of emerging REDD+ CGRs in developing countries’ contexts.

## Supplementary information

Supplementary Information

## Data Availability

The reviewed policy and project documents are listed in Appendix 1 of the Supplementary Material. Anonymized qualitative interview data can be made available upon reasonable request from the corresponding author.

## Footnotes

1 Emerson et al. (2012) use of the concept “system” here refers to the more common understanding of the interconnected environmental, social, political, and institutional conditions that influence and shape collaborative governance processes, rather than a theoretical concept.
