# Supplementary material for: Exploring System Context Contributing to Emerging REDD+ Collaborative Governance Regime in Ghana: Stakeholders Perceptions at the National Level
Source: Environ Manage. 2024 Nov 21;75(3):505–23. doi: 10.1007/s00267-024-02085-y (PMC11861400; doi:10.1007/s00267-024-02085-y)
Supplement: Supplementary file 1 — Supplementary Information [file 267_2024_2085_MOESM1_ESM.docx]

Appendix 1: Reviewed Policy Documents

1. Ghana Cocoa Marketing Board (COCOBOD). (2020). Climate-Smart Cocoa Production Standard. Prepared by the Climate Smart Cocoa Standard’s draft Committee for the Ghana Cocoa Board.
2. Cocoa and Forest Initiative (CFI). (2020). Private Sector Progress Report on *Accelerating Progress to Protect and Restore the Forests of Ghana*  (2018–2019). Accra, Ghana.
3. Government of Ghana. (2010). *Ghana REDD+* Readiness Preparation Proposal Ghana. Submitted to the Forest Carbon Partnership Facility. Accra, Ghana.
4. Government of Ghana. (2016). *Ghana REDD+ Strategy.* World Bank Forest Carbon Partnership Facility. Washington DC.
5. Government of Ghana. (2017). *Ghana Cocoa Forest REDD+ Programme. National REDD+ Secretariat, Ghana Forestry Commission and Forest Carbon Partnership Facility*. World Bank.
6. Government of Ghana. (2018). *Ghana Cocoa Forest REDD+ Emissions Reduction (ER) Programme -Resettlement Policy Framework (RPF).* Accra, Ghana.
7. Government of Ghana. (2019a). *Ghana’s First Summary of Information (SOI) - How Safeguards for REDD+ are being addressed and respected in Ghana*. Accra, Ghana.
8. Government of Ghana. (2019b). *Ghana REDD+ Social and Environmental Principles, Criteria and Indicators (PCIs)*. Accra, Ghana.
9. Government of Ghana. (2021). ER Monitoring Report (ER-MR): Ghana Cocoa Forest REDD+ Programme (GCFRP). Forest Carbon Partnership Facility (FCPF) Carbon Fund.
10. Proforest Ghana. (2021). *Engagement Principles for Ghana Cocoa Forest REDD+ Programme.* Accra, Ghana.
11. Tropenbos Ghana (2020). *TBI Working Landscapes Progress Report* (2020). Accra, Ghana.

Appendix 2: Codebook informed by the IFCG Framework

| **System context Elements** | **Context subcodes** | **Description** |
| --- | --- | --- |
| Resource conditions | - High deforestation rate - Risk of climate change - Inadequacy of future provision of forest resources | Statements related to evidence of perceived characteristics of public forest resources in the HFZ that hamper REDD+ projects from meeting their emission reduction targets. |
| Policy and legal frameworks | - Presence of legal provisions that support the multisectoral collaboration among the main relevant sectors of REDD+ such as agriculture (cocoa) and forestry | Statements related to evidence of legal frameworks and legislative regulatory systems that enable collaboration on REDD+ implementation. |
| Socioeconomic and cultural characteristics | - Densely populated region - High rate of poverty - Low educational levels - High dependence on agriculture and forest resources | Statements related to perceived socio-economic issues and the willingness to improve local communities’ livelihoods and public resource conditions at the centre of the REDD+ projects. |
| Political dynamics and power relations | - Unclarity of land tenure and responsibilities - Inequality of power and land ownership - Elite capture and high level of corruption undermining effective implementation of policies and enforcement of laws | Statements related to evidence of imbalances in power between stakeholders that use and depend on the HFZ for diverse purposes have caused people to encroach to drive deforestation and the acknowledgement of the need to tackle inequality to improve resource conditions. |
| History of Conflict | - Injustices and restrictions over access and control of forest resources - Marginalization of community members from decision-making processes. - Unmet previous local REDD+ pilot project expectations | Statements related to evidence of perceived unresolved complex conflicts and stakeholders' willingness to capacity-building efforts to address land conflicts, the rights, and benefits of local communities and other unresolved contradictions between relevant stakeholders of REDD+ to scale up such initiatives effectively. |

| **Collaboration Drivers** | **Drivers subcodes** | **Description** |
| --- | --- | --- |
| Uncertainty | - Existing perceived need to mitigate, spread, and share socioeconomic and climate change risks | - Statement related to evidence about conditions of uncertainty affecting the collaboration. |
| Interdependence | - Number of actors affected - Shared responsibilities - Perceived engagement among the stakeholders | - Statements on proof of the stated necessity to collaborate in order to progress - Statements concerning proof of earlier sector failures to solve problems or meet needs |
| Consequential incentives | - Perceived consequences of failing to engage with key actors and sectors - Perceived opportunities such as obtaining funding - Issue importance and ripeness for collaboration | - Statements about proof of positive or negative incentives that motivate leaders and participants to work together. |
| Initiating Leadership | - Presence of one or more leaders with key characteristics such as being widely respected, fair-minded, neutral, and able to support initial transaction costs for collaborative arrangements. | - Statements connected to the perceived present and identified one or more leaders who occupy a position that allows them to initiate and aid in acquiring resources to support collaborative arrangement in the initial phase. |

Appendix 3: Supplementary data

Evidence supporting the impact of five system context elements (*public resource conditions, policy and legal frameworks, socioeconomic and cultural characteristics, political dynamics and power relations, and conflict history*) on the four drivers of collaboration that influence the establishment of the GCFRP. The quotes were chosen from the analysis of interview transcripts with official project partners to qualitatively represent the diversity of perspectives revealed by respondents in this case.

| **System Context Factors** | **Quotes supporting how the system context factors influenced the adoption of a collaborative governance approach for REDD+ in Ghana’s HFZ.** |
| --- | --- |
| Resource conditions | The once-known HFZ was densely forested areas are under severe pressure from a high deforestation rate to the extent that most parts of the region have turned into [desert]. (GA-01)  Forestlands, on which REDD+ projects rely to decrease emissions, are also experiencing considerable deforestation. A collaborative REDD+ initiative is required to address the complex and interconnected concerns of deforestation and climate change, utilizing the strengths of many stakeholders, and ensuring the long-term viability of conservation activities (EN- 02).  The forest resources within Ghana’s High Forest Zone (HFZ) persistently confront significant challenges posed by deforestation and climate change, both of which are global issues demanding collective action. Initiatives such as REDD+ projects play a vital role in addressing these challenges by efficiently reducing emissions at a reasonable cost. These projects engage diverse stakeholders who collectively bear the responsibility for protecting and preserving forests. The imperative for collaboration is underscored as essential for ensuring the success and long-term sustainability of such initiatives (CSO-01).  Addressing deforestation requires a collective effort involving diverse stakeholders to balance the needs of people and the environment. Collaboration, along with sustainable practices and policies, is crucial for the long-term conservation of forests (PS-01). |
| Policy and legal frameworks | I think we had a lot of legislative contexts particularly Ghana’s National REDD+ Strategy, which was launched in November 2016, called for multisectoral collaboration and coordination among decision-making organizations in the agricultural and forest sectors to address the direct and indirect drivers of deforestation and forest degradation in the High Forest Zone (GA– 03).  The Ghana Cocoa Forest REDD+ Program is firmly established in a highly supportive policy context. Recently, Ghana has adopted crucial policies such as the National Climate Change Policy (2013) and the National Forest and Wildlife Policy (2012), laying a solid foundation for the effective implementation of REDD+ (EN – 03).  Ghana is a participant in the global effort of the United Nations Framework Convention on Climate Change (UNFCCC), working towards addressing climate change and its consequences through collaborative efforts with stakeholders. The Ghana Cocoa Forest REDD+ program, an initiative formed in conjunction with the UNFCCC program, exemplifies the country's commitment to this endeavour (PS-01).  As one of the countries engaged in the Paris Agreement, Ghana actively participates in a renewed framework for global cooperation on climate action. The emphasis is on joint efforts to fulfil Nationally Determined Contributions (NDCs). The collaborative REDD+ project is instrumental in aligning with Ghana's outlined NDCs in the Paris Agreement (CSO-02) |
| Socioeconomic and cultural characteristics | Apart from the rising growth in the rural population, a significant hindrance to sustainable forestland management is attributed to insufficient awareness of enhanced farming techniques and a lack of education (GA-04).  Poverty and a lack of alternative sources of livelihood are direct causes of intensive deforestation and degradation of ecosystem services in the HFZ, which international climate finance from REDD+ projects can help to address and reduce emissions cost-effectively (CSO-01).  The high dependence on forest resources and the ongoing expansion of cocoa cultivation into forested regions persist as significant threats to biodiversity in the area. Despite existing government legislation and support, they do not offer sufficient motivation to curb ongoing deforestation and degradation in the HFZ. Collaborative efforts allow for a more comprehensive and holistic approach to address the multifaceted nature of these challenges (PS-01).  The primary economic activities in the HFZ include agriculture, specifically cocoa farming, and the extraction of non-timber forest products (NTFPs) for subsistence. However, the cocoa sector is at considerable risk from climate change, affecting various stakeholders in the cocoa value chain (EN-01). |
| Political dynamics and power relations | Land tenure and carbon rights remain unclear and contested, with unresolved contradictions and overlapping claims of rights between different departments of government and customary forms of land ownership on the ground (CSO-02).  There is profound concern about the continued marginalization of local communities from decision-making processes affecting the forests on which they depend. When local voices are not heard, we see a direct correlation with increased rates of deforestation (EN-02).  We’ve seen several instances where access to and control of natural resources is politically mediated, where powerful politicians, private players, and corporations have had significant influence on land-use decisions, logging activities, and the deforestation process in the HFZ. Collaborative governance is urgently needed to reorient strategies to address unequal power relations by fostering inclusive decision-making and a more equitable and sustainable approach to resource management that considers all stakeholders’ interests in the fight against deforestation (PS-01).  Political patronage and corruption have a long history in Ghanaian forest management, resulting in favouritism toward certain stakeholders, unequal distribution of benefits, and a lack of transparency in decision-making processes (GA-01).  The existing governmental policies favour commercial logging and plantations without taking into consideration the needs and perspectives of local communities. The continued disregard of community land tenure rights leads to conflicts over the sustainable use and management of forestlands (EN-03). |
| History of conflicts | Ghana contends with an intricate framework of land and forest tenure characterized by legal pluralism. The management authority over agricultural land and forests lacks clarity, leading to confusion regarding the jurisdiction of traditional leaders versus state management and community rights. Rebuilding trust among stakeholders proved challenging, necessitating concerted coordinated and collaborative efforts (EN-02).  The complexity of land ownership structures hampers efforts to combat deforestation, leading to conflicting management goals, uncertainty, power imbalances, and unsustainable land use. Successful collaboration is vital to consider all perspectives for comprehensive and legitimate solutions (CSO-01).  A significant conflict of interest arises regarding forest lands given that a significant portion of the local population is engaged in agriculture particularly the cultivation of cocoa as a cash crop and are keen on expanding agricultural areas (GA-03).  Historical land tenure conflicts are intensified by excluding community members from comprehensive project participation and failing to meet high project expectations. To mitigate these issues, it is crucial to prioritize the rights, livelihoods, and benefits of local communities by placing them at the core of REDD+ projects (PS-01).  Tackling tenure conflicts is the first step if we can win the war against deforestation. It requires more than just political party manifestos and sweet slogans; it requires proactive participation from private and local community stakeholders (CSO-03). |
